# Technology-integrated nursing interventions to improve adherence to tuberculosis medication: a scoping review

**DOI:** 10.1186/s12912-025-03796-1

**Published:** 2025-09-02

**Authors:** Eli Indawati, Cecep Eli Kosasih, Hartiah Haroen, Anastasia Anna

**Affiliations:** 1 Faculty of Nursing, Universitas Padjadjaran, Sumedang, Jawa Barat Indonesia; 2 Department of Critical Care Nursing, Faculty of Nursing, Universitas Padjadjaran, Sumedang, West Java 45363 Indonesia; 3 Department of Community Nursing, Faculty of Nursing, Universitas Padjadjaran, Sumedang, West Java 45363 Indonesia

**Keywords:** Digital health, Medication adherence, Nursing intervention, Tuberculosis

## Abstract

**Background:**

Despite global efforts, adherence to tuberculosis (TB) treatment remains suboptimal. Nurses play a crucial role in supporting treatment adherence through direct care and the integration of digital health tools. Nursing interventions utilizing technology have great potential to enhance medication adherence by providing education, reminders, and remote monitoring tailored to patient needs.

**Objective:**

To explore nursing interventions involving technology that improve medication adherence among TB patients.

**Methods:**

This scoping review followed the Arksey and O’Malley framework. Literature was systematically searched through Scopus, PubMed, and Web of Science using keywords such as “nursing intervention,” “tuberculosis,” and “medication adherence.” Inclusion criteria encompassed studies published within the last ten years, involving people with TB, and describing technology-integrated nursing interventions aimed at improving treatment adherence. A total of 12 studies were included and thematically analyzed using a descriptive qualitative approach with NVivo software.

**Results:**

Five main themes were identified: (1) The effectiveness of digital technology in improving medication adherence, (2) Limitations in access to healthcare services and the role of technology as a solution, (3) Video technology for directly observed therapy (VDOT), (4) Interactive reminder system (Two-Way SMS), and (5) Patient motivation in adhering to TB treatment through digital technology. Nurses were central to assessing patients’ needs, training them to use digital tools, and maintaining adherence through follow-up and education.

**Conclusion:**

Nursing interventions that incorporate digital technology, such as SMS reminders, VDOT, and mobile health applications are effective in supporting medication adherence among TB patients. These tools empower nurses to extend care beyond the clinical setting, particularly in underserved areas.

**Supplementary Information:**

The online version contains supplementary material available at 10.1186/s12912-025-03796-1.

## Introduction

Despite global efforts, TB treatment adherence is still low, in developing countries drug adherence is 40%, in India 60.8% of patients are adherent to TB treatment regimens, in Southern Ethiopia, the prevalence of non-adherence was found to be 24.5%, mainly due to sociodemographic factors, patient age, family size, family history, and smoking habits [1]. According to the World Health Organization (WHO), Global Tuberculosis Report 2024, there are approximately 10.8 million new cases of TB, mainly in low- and middle-income countries, with limited health care resources. Based on WHO data for 2024, TB disease annually occurs in 30 countries, five of which are: India (26%), Indonesia (10%), China (6.8%), Philippines (6.8%) and Pakistan (6.3%). The disease causes approximately 1.6 million deaths worldwide [2]. India has the highest number of cases, with more than 2.6 million new cases annually, accounting for about 26% of the global total [3]. China records around 680,000 new cases of TB per year, while Indonesia faces around 969,000 cases, placing it among the countries with the highest incidence in Southeast Asia [2].

Tuberculosis (TB) has a significant impact on public health, covering medical, social, and economic aspects. TB can cause serious damage to the lungs and other organs and lead to serious complications, especially in individuals with weakened immune systems, including those with HIV/AIDS, which increases the risk of drug-resistant TB (MDR-TB) [3]. From a social perspective, stigmatization of TB often leads to social exclusion, which discourages patients from seeking treatment or continuing therapy, exacerbates the spread of the disease, and causes psychological effects, such as anxiety and depression [4]. Economically, people with TB often lose productivity due to being unable to work during the long treatment period, resulting in loss of income and financial instability [5]. At the community level, TB control burdens national health systems and hampers economic growth, especially in high-prevalence countries, due to increased treatment costs and reduced productivity [6].

Tuberculosis (TB) treatment faces a number of complex challenges. The standard TB treatment regimen involves a combination of drugs that must be taken regularly for at least 6 months [7]. The long duration of therapy and complex regimens often lead to non-compliance, especially in patients who feel their symptoms improve before therapy is completed. In addition, TB treatment can cause side effects, such as nausea, headache, hepatotoxicity, and neuropathy, which further increases the risk of discontinuation of therapy by patients [8]. Non-adherence to treatment has serious implications, including the emergence of multidrug-resistant TB (MDR-TB), which requires longer and much more expensive treatment, with lower treatment success rates. Research shows that patient adherence to TB treatment varies, with some studies reporting non-adherence rates as high as 20–30% in some high TB burden countries [9].

Treatment adherence in TB patients is influenced by various interrelated factors. One of the main factors is the length of treatment, which often makes patients feel tired and reluctant to continue therapy [10]. In addition, complex treatment regimens and drug side effects such as nausea, dizziness, or liver damage can decrease patients’ motivation to adhere to treatment [11]. Social factors also play an important role; the social stigma associated with TB can discourage patients from being open about their disease and hinder their access to health services. Economic factors, such as the cost of traveling to health facilities or loss of income due to time spent on treatment, can be significant barriers, especially in low-income countries [12]. Lack of involvement of health workers in providing adequate support and education can also lead to patients not understanding the importance of complying with therapy until completion. Psychological factors, such as the patient’s level of anxiety, depression, or internal motivation, as well as support from family and the surrounding community, also affect the level of adherence of TB patients to treatment [13].

In the context of tuberculosis treatment, nurses serve as key implementers of adherence interventions, not only by delivering medication but also by leveraging digital tools to enhance patient education, monitor progress, and maintain motivation throughout the treatment journey. Nursing interventions play an important role in improving treatment adherence in people with TB. Digital-based counseling is important to be implemented by nurses to improve medication adherence in tuberculosis (TB) patients [14]. In fact, this approach has great potential to overcome the challenges of TB treatment. Various nursing strategies have been implemented, such as counseling to provide an understanding of the importance of completing treatment, as well as skills training to help patients cope with drug side effects and how to manage daily medication [15]. Although counseling is an essential nursing intervention, in the reviewed studies it was often delivered through digital platforms such as SMS, video calls, or mobile apps, rather than as a stand-alone service. In addition, medication reminders, either in the form of a recorded schedule or direct medication administration by a nurse, are also used to ensure that patients follow their medication regimen appropriately [16]. An innovative nursing intervention approach that is gaining popularity is the use of digital technology, includes various digital adherence technologies (DATs) such as mobile apps, electronic medication monitors (EMMs), video-observed therapy (VOT), and short message service (SMS) reminders. such as mobile apps or SMS reminders, which can help patients remember their medication schedule and monitor their progress. Several studies have shown that these technology-based interventions are effective in improving medication adherence [17–20].

Nursing interventions utilizing technology have great potential to improve tuberculosis medication adherence [17]. Technology-based interventions allow for more efficient monitoring, easier communication, broader education, more personalized support, and the effectiveness of technology-based interventions can be integrated into the health care system [16]. Although nursing interventions involving technology offer great potential to improve TB medication adherence, there is still a gap between the potential and the reality of implementation in the field due to various factors such as access to technological infrastructure, patient reluctance, lack of family support so that integrated nursing interventions are needed.

Previous review have shown that treatment adherence is important for improving the recovery of people with TB [21]. However, no reviews have described interventions by nurses to improve treatment adherence in people with TB. Meanwhile, nurses have important roles as educators, counselors, and advocates in addressing problems in people with TB [22, 23]. Therefore, this scoping review aims to explore various technology-integrated nursing interventions that have been implemented to improve medication adherence in people with tuberculosis (TB), identify the most effective technology-based strategies facilitated by nurses, and provide a clearer understanding of approaches that require further development. The findings of this study are expected to guide nursing practitioners in designing and implementing more effective, evidence-based, and technology-enhanced interventions to support TB treatment adherence.

## Methods

### Study design

This study used a scoping review design with the Arksey & O’Malley approach designed to map various nursing interventions in improving treatment adherence in people with TB [24]. This approach was chosen because scoping reviews allow for a broad and comprehensive exploration of the different types of interventions that have been implemented in various contexts and can cover a wider range of literature. The steps in this scoping review include: first, identification and selection of a clear topic or research question; second, selection and screening of relevant literature using well-defined inclusion and exclusion criteria; third, extraction of data from the selected articles; fourth, reporting the results in the form of relevant themes or categories. With this approach, this study aims to provide a comprehensive picture of the various nursing interventions that have been implemented in improving adherence in people with TB, as well as provide directions for further research. This research used Nvivo 15.2 software ID: d338db5ba8d4.

### Search strategy and eligibility criteria

The literature search was conducted in three major databases: Scopus, PubMed, and Web of Science, selected for their reliability in providing medical and nursing literature and their broad coverage of global health research. Keywords combined both general terms and MeSH (Medical Subject Headings), including “nursing intervention,” “medication adherence,” “tuberculosis,” and “patient compliance.” Boolean operators (AND, OR, NOT) were applied to refine the search, such as “nursing intervention AND medication adherence AND tuberculosis,” and filters were used to ensure the quality and relevance of the selected articles.

**Fig. 1:**
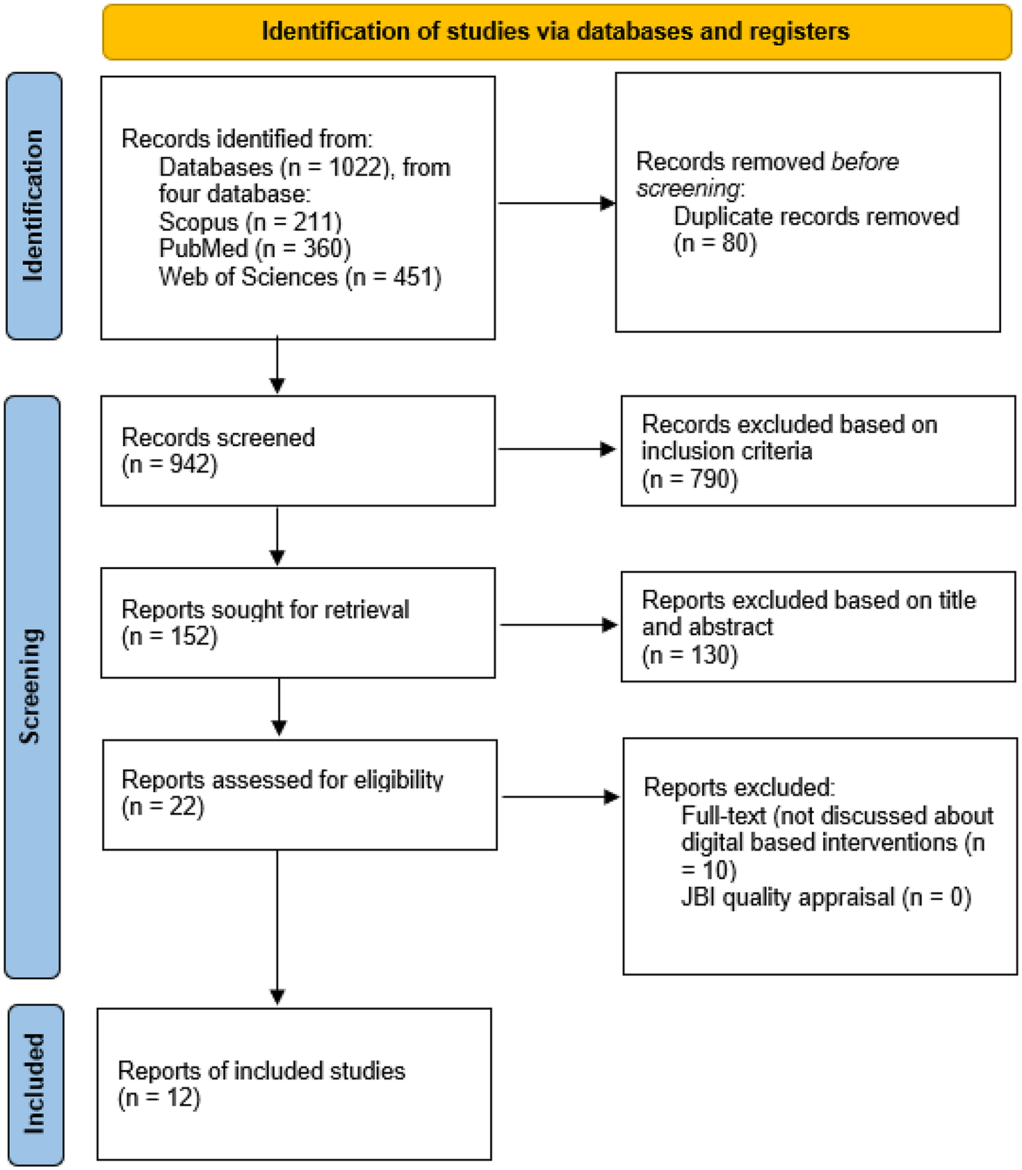
PRISMA flowchart

In this review, *nursing interventions* were defined as actions or strategies in which nurses played a direct or facilitative role in planning, implementing, or monitoring interventions to improve TB medication adherence. These could include direct care activities, such as counseling, patient education, or medication administration, as well as indirect approaches using digital tools—SMS reminders, mobile health applications, or video-based observed therapy. Interventions carried out exclusively by other health professionals were excluded unless nurses were significantly involved in delivering or coordinating the intervention. The article search reported using the PRISMA Flowchart to illustrate the article selection process, from article identification, sorting based on eligibility criteria, to articles included in the analysis (Fig. 1).

### Inclusion and exclusion criteria

Articles were included if they met the PCC framework: the population was individuals diagnosed with active tuberculosis or undergoing TB therapy; the concept was nursing interventions, particularly those integrating technology, aimed at improving treatment adherence; and the context was TB care provided in healthcare facilities or community-based settings. Only experimental designs (RCT or quasi-experimental) published in English or Indonesian between 2014 and 2024 were considered, to ensure that the findings reflected current TB management guidelines and the growing adoption of digital health interventions. Studies were excluded if they were not directly related to TB or medication adherence, if they did not describe a specific nursing intervention, or if they lacked measurable adherence outcomes. Qualitative or survey-based studies without a clear intervention component, as well as articles with insufficient detail on the nurse’s role in the intervention, were also excluded.

### Data extraction

Data extraction will be conducted by two authors independently to minimize bias and ensure objectivity. The data to be extracted includes information about the author, the purpose of the study, the study design, the characteristics of the sample (including the number, type, and age of participants), the country where the study was conducted, the instruments used (such as questionnaires or interviews), the type of nursing intervention applied, and the outcomes reported. The data extraction process will be carried out using manual tables prepared to facilitate comparison between studies. If there are differences of opinion between the two authors in the extraction process, a third author who is an expert in the field will be invited to provide clarification and reach agreement. This will ensure the accuracy and completeness of the extracted data. The data extractions are listed in Table 1 below (Table 1):

**Table 1:** Extraction data

No	References	Aim	Country	Sample	Design	Interventions	Result
1.	[1]	Evaluate if MERM-observed therapy improves HRQoL and reduces costs compared to DOT in TB patients.	Ethiopia	109 adults with new drug-sensitive pulmonary TB	RCT	Participants were trained to use the MERM device, received a 15-day TB medication supply, and returned to the clinic biweekly for tablet checks and data reviews. Missed doses were discussed with participants. The EQ-5D-5 L tool was used.	This study found that MERM device-observed self-administered therapy for TB patients improved HRQoL and reduced catastrophic costs compared to standard DOT. Patient-centered digital health technologies may help address structural barriers to anti-TB therapy.
2.	[2]	Assess the effectiveness of SMS reminders on treatment adherence and cure rates in TB patients.	Cameroon	260 patients with tuberculosis	RCT	Patients in the intervention group received free daily SMS reminders in French for six months. A follow-up call ensured the patient received and understood the initial welcome SMS. The SMS content, developed by the research team, aimed to remind and motivate patients to take their anti-tuberculosis medications. Messages were updated every two weeks to sustain patient engagement.	Out of 279 participants, treatment success at five months was 81% in the intervention group and 74.6% in the control group (*p* = 0.203). At six months, cure rates were 63.5% and 62%, respectively (*p* = 0.791). Dropout rates were similar (34.3% vs. 32.4%), with no significant differences in secondary outcomes. Both groups showed high satisfaction with patient management (~ 99%).
3.	[3]	Measure the impact of Zindagi SMS on treatment success for drug-sensitive TB.	Pakistan	Patients with positive pulmonary tuberculosis who were on treatment for less than two weeks;	RCT	Zindagi SMS sent daily reminders and motivational messages in Urdu to encourage patient engagement. Patients responded via SMS or missed calls to confirm medication intake. They received PKR 60/month for response costs, initially at clinics and later via phone transfers.	There was no significant difference in treatment success between the Zindagi SMS and control groups (83% vs. 83%, *p* = 0.782). Additionally, the program had no effect on self-reported medication adherence during unannounced visits.
4.	[4]	Investigate the role of SMS in enhancing TB treatment adherence and health awareness.	China	350 patients tuberculosis	RCT	Patients in the SMS group received daily reminders throughout treatment, while the control group followed conventional DOT. SMS content included reminders to take medicine on time, follow up with reexaminations, practice hygiene, and maintain a healthy lifestyle.	The SMS group had a significantly higher treatment completion rate (96.25%) compared to the control group (86.84%) (*p* = 0.002). The SMS group also had lower interrupted treatment and missed dose rates (*p* = 0.001; *p* < 0.001).
5.	[5]	Compare ingestion detection accuracy and medication adherence using WOT versus DOT in TB treatment.	USA	61 patients with tuberculosis	RCT	Stage 1 (2–3 weeks) involved training on WOT use and patch changes. In Stage 2, participants changed patches weekly, monitored medication logs, and received reminders if doses were missed. Follow-up visits were every 2 weeks, then monthly. WOT users preferred continuing the treatment until completion, extending the study duration.	WOT confirmed 93% of doses, significantly higher than DOT at 63% (*p* < 0.001). Excluding non-working days, WOT showed 95.6% adherence, similar to DOT’s 92.7% (*p* = 0.31). All participants preferred WOT, with less than 10% experiencing minor skin issues.
6.	[6]	Evaluate the implementation of VDOT and its impact on treatment completion for patients on 3HP.	USA	71 patients with tuberculosis	RCT	Patients experiencing side effects during VDOT sessions were contacted for follow-up and managed accordingly. If patients missed their scheduled appointment, they were contacted by phone within 30 min, and a voicemail or SMS was sent to reschedule. SMS reminders for appointments were sent with patient consent, in line with NYC DOHMH policy.	Of the eligible patients, 70% were assigned to V3HP, with an 88% treatment completion rate, compared to 64.9% in the 3HP clinic DOT group (*P* < 0.001). A total of 360 video sessions were conducted, with a median of 8 sessions per patient and 4 min per session. Adherence issues occurred 104 times, but no major side effects were reported.
7.	[7]	Determine the effectiveness of VDOT compared to in-person DOT for anti-TB treatment.	USA	390 patients with tuberculosis	RCT	Patients showed and named each pill before swallowing it, confirming ingestion by opening their mouths. Side effects prompted a physician consultation. Sessions were documented in the EMR, with missed appointments followed up by phone or home visits.	Adherence to VDOT sessions was 95% (3292/3455) compared to 91% for in-person DOT (32,204/35,442, *P* < 0.01). VDOT allowed DOT workers to observe up to 25 patients daily, similar to clinic-based DOT workers, but double the number for community-based workers (*n* = 12). Treatment completion rates were similar for VDOT and in-person DOT (96% vs. 97%, *P* = 0.63).
8.	[8]	Test whether VOT improves treatment observation levels.	USA	383 patients with tuberculosis	RCT	Patients recorded daily doses via video for VOT, with remote observation three to five times a week. Side effects were also reported in the videos. Smartphones and data plans were provided by the study, and patients agreed to return the phones at the end of treatment.	70% of VOT patients completed ≥ 80% of scheduled observations, compared to 31% of DOT patients (OR 5.48, *p* < 0.0001). In a restricted analysis, 77% of VOT patients achieved the primary outcome, versus 63% of DOT patients (OR 2.52, *p* = 0.017). Adverse events, mainly stomach pain, nausea, and vomiting, were reported more frequently in VOT patients (14%) than in DOT patients (8%).
9.	[9]	Compare treatment completion and safety of self-administered versus directly observed isoniazid and rifapentine.	United States, Spain, Hong Kong, and South Africa.	1002 adults (aged ≥ 18 years) recommended for treatment of latent tuberculosis infection.	RCT	Participants received once-weekly isoniazid and rifapentine through direct observation, self-administration with monthly monitoring, or self-administration with weekly text reminders and monthly monitoring.	Treatment completion rates were 87.2% for direct observation, 74.0% for self-administration, and 76.4% for self-administration with reminders. In the U.S., completion rates were 85.4%, 77.9%, and 76.7%, respectively.
10.	[10]	Assess the impact of text messaging and medication monitors on TB medication adherence.	China	300 active pulmonary TB patients	RCT	Patients received medication reminders via text or audio, with follow-up messages if needed. Missed doses were tracked by pill count, SMS replies, or monitor openings.	The percentage of patient-months with at least 20% missed doses was 29.9% in the control group, 27.3% in the text messaging group, 17.0% in the medication monitor group, and 13.9% in the combined group.
11.	[11]	Evaluate the role of mobile SMS reminders in improving drug compliance in DOTS patients.	Pakistan	148 patients with tuberculosis	RCT	The intervention group received monthly visits for drug collection and daily SMS reminders during the first 2 months of treatment. The SMS included a message in Urdu and an illustration for illiterate patients. The control group only received monthly visits without SMS reminders.	Treatment default occurred in 7 patients (4.7%), with 3 patients (4.1%) in the intervention group and 4 patients (5.4%) in the control group. No significant difference was found between the groups (*p* = 0.983).
12.	[12]	Assess the effectiveness of an mHealth package on medication adherence in TB patients on DOTS treatment.	India	220 adult TB patients	Quasi‑experimental study	The mHealth package included daily text messages and weekly 10-minute phone calls for 90 days, providing medication reminders and addressing side effects. It was pretested with 25 TB patients in Delhi to ensure comprehension.	Occupational interference and forgetfulness were the main reasons for medication nonadherence. In the intervention group, adherence to the daily DOTS regimen increased from 85.5% at baseline to 96.4% post-intervention (*P* = 0.004), while no significant change was observed in the control group (*P* = 0.328).

### Quality assessment

Quality assessment of articles included in this review will be conducted using the Joanna Briggs Institute (JBI) Quality Appraisal. The JBI assessment is a tool used to evaluate the methodological quality of studies, by assessing the study design. For RCT studies, the JBI assessment consists of 13 statements that measure various aspects of quality, including randomization, bias control, and sample size. For quasi-experimental studies, the JBI assessment consists of 9 statements focusing on variable control and internal validity. The quality of the article will be assessed by two authors independently, and in case of disagreement, a third competent author will be engaged to resolve the discrepancy. Articles that score more than 70% on the JBI assessment will be considered to meet sufficient quality standards to be included in the analysis, If there is low quality, it will still be included but discussed critically.

### Data analysis

Data analysis in this scoping review was conducted using a conventional content analysis approach, which is suitable for inductively identifying and synthesizing themes from the literature. This method was chosen to allow themes to emerge directly from the data without relying on pre-existing theoretical frameworks. The analysis focused on the Results section of the included studies, from which key information regarding nursing interventions and their effect on treatment adherence in TB patients was extracted.

Thematic analysis of qualitative data according to Virginia Braun and Victoria Clark (2006), consists of 6 steps: (1) familiarize with the data, (2) code, (3) look for themes, (4) review themes, (5) define themes and name themes, (6) write a report.

The extracted data were read repeatedly by two independent reviewers to ensure familiarity with the content, followed by open coding to identify important statements and recurring ideas. These codes were then grouped into categories reflecting intervention type, implementation strategy and contextual influences. Through this inductive process, six main themes were developed that represented patterns across the studies reviewed, defining the themes and providing themes, the next stage of compiling the report.

To ensure credibility and consistency of findings, coding and categorization were conducted independently by two reviewers. Discrepancies were resolved through discussion with a third reviewer until consensus was reached. This approach allowed the review to comprehensively map the scope of nursing interventions, clarify key concepts, and identify research gaps and emerging trends in the field. This research also uses Nvivo 15.2 software ID: d338db5ba8d4. The results of the Nvivo code can be seen in G.2 below:

G 2. Code analysis results


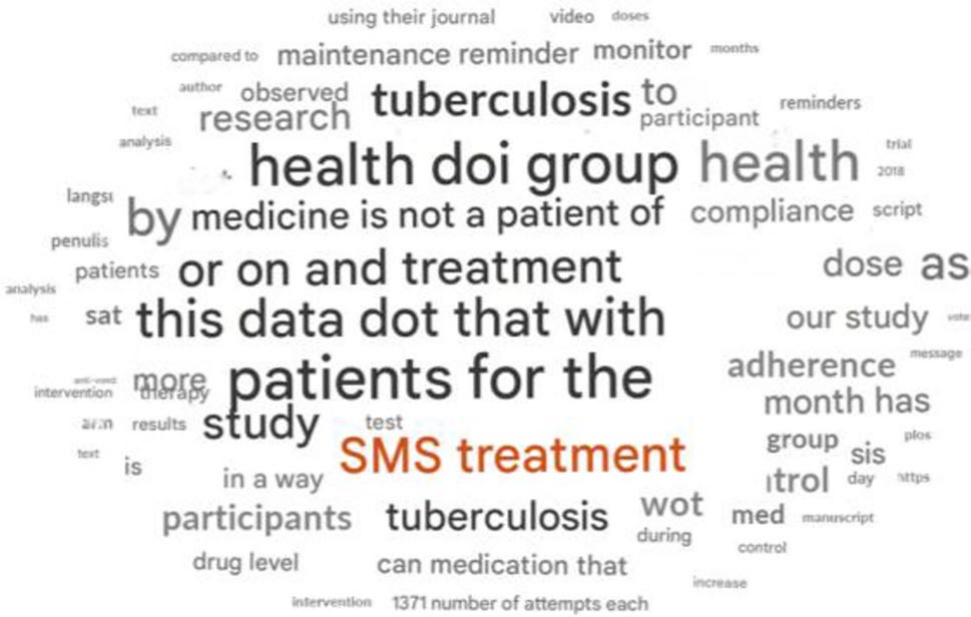


## Results

Based on initial research searches from three databases, the author found 1022 reports. The results of article duplication using Mendeley, obtained 80 duplicate articles. The results of elimination using inclusion criteria by two authors obtained 790 articles. Then, the author eliminated using the title and abstract, 130 articles were obtained that were not in accordance with the research objectives. After that, the author read the full-text articles and obtained 12 articles that discussed nursing interventions to improve treatment adherence in tuberculosis patients. All articles have been quality assess, all articles have a JBI assessment score above 70%.

This scoping review analyzed a total of 12 articles that addressed nursing interventions to improve treatment adherence in tuberculosis patients. The research design of these articles included 11 RCT studies and one quasi-experimental study. The RCT study contained triple-blind performance bias and 1 article with a small sample size of 71 people. The articles came from various countries, namely Ethiopia, Cameroon, Pakistan, China, the United States, Spain, Hong Kong, South Africa, and India, with details of one article each from Ethiopia, Cameroon, Pakistan, China, and Spain, two articles from the United States, and one article involving several countries (the United States, Spain, Hong Kong, and South Africa). The number of respondents involved in these articles varied, with the range of respondents ranging from 61 to 1002 tuberculosis patients.

### Theme 1: Effectiveness of digital technology in improving medication adherence

Several articles have demonstrated the use of technology-based interventions as an effective approach in improving TB treatment adherence. The use of video reminders significantly helped patients in remote areas to adhere to their treatment schedule [23]. Patients record themselves taking medication and send the video to the nurse for verification, allowing the nurse to monitor medication adherence in real time. In addition, the use of technology-based applications that provide automated reminders has been shown to help patients become more organized in their TB treatment [24]. In addition, this study revealed the importance of using technology-based applications that provide ongoing support to patients, improve communication between patients and healthcare workers, and detect medication non-adherence early [26]. Technology-based reminder systems have also yielded significant results in improving therapy completion in more complex TB patients [27, 28].

### Theme 2: Limited access to healthcare and technology as a solution

The role of nurses is crucial in designing innovative and adaptive interventions with a focus on utilizing technology as a solution to TB medication adherence, by identifying and thoroughly assessing the patient including the patient’s clinical condition, family and environmental support, geographical, socioeconomic, and digital literacy. The biggest challenge in TB treatment is limited access to health services, especially in remote areas. This study shows how SMS-based reminders can help address this issue in a more affordable and accessible way across neighborhoods without the need for a large health infrastructure [23]. Santos et al. (2024) (Article 3) added that SMS reminders have a positive impact, especially for patients in rural areas who have limited access to health services. The use of technology allows patients to access health information and medication reminders even though they live far from health facilities. It also emphasizes the importance of technology-based applications in facilitating treatment monitoring of patients in hard-to-reach areas [29].

### Theme 3: Video technology for observation of treatment (VDOT)

Nurses act as central facilitators in VDOT implementation. Their roles include providing initial training, instructing patients and treatment supporters (PMOs) on video use, and following up on missed doses or reported side effects. Video Directly Observed Treatment (VDOT) creates a flexible yet effective method of observation, the nurse can ensure the patient swallows every dose of medication as prescribed, the role of the nurse is the main facilitator and monitor of the VDOT process by explaining and training the use of technology to the patient and the medication supervisor (PMO), how to use the selected video application or platform (e.g., WhatsApp Video Call, Zoom, or a dedicated VDOT application), teach how to record a video (if asynchronous) or start a video call (if synchronous). Video technology is used to monitor treatment adherence through virtual observation. VDOT is effective in improving TB treatment completion, although there are several technical and social challenges that must be overcome to maximize its benefits [30–33]. The use of VDOT allows patients to remain at home, reducing reliance on hospital visits and saving time and costs [28]. However, there are still challenges related to internet connection and technical capabilities of some patients.

### Theme 4: Interactive reminder system (Two-way SMS)

Technology-based nursing interventions through automated text messages (SMS reminders) of medication reminders and motivation at regular intervals, nurses can monitor patients taking medication through text messages (SMS) and simple reminder automated phone calls to patients’ cell phones but are more effective and efficient in patients who do not have smartphones.Some studies emphasize the use of interactive SMS reminder systems, which allow patients to confirm their adherence through message replies or phone calls [19, 21, 23]. Two-way SMS systems can increase patient involvement in their treatment and help the medical team monitor their progress [20, 24, 31]. The use of SMS to remind patients about their treatment is a strategy that has been widely explored in these studies. Regular SMS messaging can improve TB treatment completion rates and help patients adhere to their treatment schedule [24, 29]. Two-way SMS-based reminders, which improve adherence and allow patients to confirm whether they have taken their medication as scheduled [27]. Two-way interactions can improve communication between patients and healthcare providers, facilitate the provision of needed support, and improve patients’ adherence to their medication [34].

Nursing interventions in long-term treatment the role of nurses can conduct an initial assessment in assessing patient needs in determining the type of technology based on digital literacy, patient technology access, and personal preferences. The impact of technology-based reminders can improve medication adherence, prevent drug withdrawal, patient and family empowerment, and resource efficiency. The reviewed articles show that technology-based reminders such as SMS and digital apps play an important role in long-term TB treatment. Technology-based applications allow patients to monitor and report treatment status, while automated reminders reduce the risk of non-adherence [29]. Technology-based reminders have a positive impact on long-term patient adherence, by increasing patient engagement in their treatment [35]. Based on age in the summarized studies, the preference for the SMS method is shown in Table 2 below (Table 2):

**Table 2:** Preference for SMS method based on age

No	Group	Age	SD
1	Intervention	47–59	13,42
2	Control	34–60	1,00

Many articles highlight the importance of sustainability and patient preference in choosing technology for medication reminders. Further research shows that patients prefer to use technologies that allow self-monitoring of medication, such as the use of WOT (Wireless Observed Therapy) or VDOT [30–32]. This study emphasizes the importance of choosing a technology that is appropriate for the patient’s condition and their preferences, as factors such as age, education, and technological ability can affect the success of the intervention [33].

### Theme 5: Patient motivation to adhere to TB treatment through digital technology

Nurses can leverage digital technology with a planned and adaptive approach to actively monitor adherence and build and maintain motivation in people with TB, through innovative and creative interactive educational interventions that are more personalized, thereby increasing motivation to take medication. Patient motivation plays an important role in successful TB treatment. The use of technology-based applications that provide personalized reminders has been shown to increase patient motivation to remain committed to treatment [24]. The reminder videos sent at regular intervals serve not only as reminders, but also as visual motivators that remind patients of the importance of regular medication [23].

## Discussion

The results of this scoping review show that there are 12 articles that discuss nursing interventions to improve adherence to taking medication in tuberculosis (TB). Adherence to medication in people with TB is an important factor in achieving recovery and preventing the spread of the disease. Adherence to treatment is often hampered by various factors, such as drug side effects, cost of treatment, limited access to health facilities, and lack of patient understanding of the importance of continuous treatment [36]. If patients do not adhere to the recommended treatment regimen, the risk of developing drug resistance increases, which can worsen the condition and increase the potential for transmission of TB to others [37].

The differences in results in this scoping review are also influenced by differences between developed and developing countries. Previous studies have shown more positive results regarding the use of technology in improving TB treatment adherence, with better technological infrastructure and easier access to health services. Nursing interventions should be tailored to the patient’s condition and needs for long-term treatment success [38]. The use of technology-based reminders, such as SMS and apps, has been shown to be effective in improving adherence especially living in remote areas. Previous research has shown that technology, especially SMS, can increase patient engagement in their treatment in an accessible and cost-effective way [38].

Research from developing countries has additional challenges, such as limited access to technology and health services, which may affect the effectiveness of interventions [38]. However, studies in developing countries show that the use of SMS-based reminders or digital apps still has a positive impact, despite challenges related to limited infrastructure and digital literacy [39].

Nursing interventions through direct interaction via messaging allow medical staff to monitor adherence more actively and provide the support patients need, such as answering questions or providing motivation [40]. While technology can address access issues, social and economic factors, such as difficulty understanding or using technology, remain a challenge that needs to be addressed, especially in areas with low levels of education and technology skills [41]. The use of interactive SMS, has been shown to increase patient engagement in tuberculosis treatment. This two-way SMS system not only serves as a reminder, but also as a tool to improve communication between patients and healthcare workers [42]. This finding is consistent with previous studies showing that more open and responsive communication between patients and healthcare providers can increase patients’ sense of responsibility for their treatment [43]. With automated reminders, patients can more easily remember the time and dose of medication, even in busy or difficult situations. However, it is important to note that the effectiveness of these reminders may vary depending on the level of patient acceptance and preference of the technology used [44]. This study also suggests that the long-term sustainability of technology-based reminders requires continuous evaluation to adapt to the evolving needs of patients and their changing preferences for the type of technology used [45].

Research shows that digital technology not only serves as a medication reminder, but can also act as an effective motivational tool for patients. Video reminders and technology-based apps can provide an additional motivational boost for patients to keep up with their treatment.” [46]. Patients tend to be more disciplined in following therapy, as they feel a sense of responsibility to fulfill with regular reminders. It also gives patients a sense of control over their own treatment, which in turn improves adherence [47]. In addition, the use of more interactive technologies, such as apps that allow patients to track their progress, provides a sense of accomplishment and increases motivation to complete treatment [48].

The use of technology-based reminders enables more flexible and affordable treatment monitoring, and increases patient engagement in their treatment process. In nursing practice, these results suggest that technology-based interventions can be integrated as part of TB treatment management strategies [49]. The results showed that the use of video technology (VDOT) was effective in improving TB treatment adherence. VDOT allows patients to undergo treatment at home, reduces the need for hospital visits, and facilitates monitoring by medical personnel [50]. Previous studies have shown that VDOT can improve medication adherence, especially for patients who have difficulty accessing health facilities regularly. However, technical challenges such as video quality and unstable internet connection are the main obstacles in the implementation of such technology [51]. In developing countries, the problem of cost, low technology settings, patient refusal, Limited access to adequate technology and infrastructure can exacerbate this problem, whereas in developed countries, the use of this technology is more accessible, but still requires careful management to ensure its success [52, 52].

Nurses in resource-limited settings should prioritize SMS-based reminders over VDOT due to lower infrastructure requirements. Training programs for nurses on digital health tools should be integrated into TB management guidelines. Technology-based adherence reminders from 12 articles on TB medication adherence reminders, results in Table 3 (Table 3):

**Table 3:** Tech-based compliance reminders

No	Adherence reminder	Result
1	SMS	96.25% in 4/6 studies
2	VOT	79.00% in 1/1 study
3	VDOT	95.00% in 1/2 studies
4	WOT	93.00% in 1/1 study
5	mHealth	96.04% in 1/2 studies

Generally, there’s a tendency for older patients with low technological literacy to prefer SMS-based reminders due to their simplicity and accessibility. Meanwhile, younger patients with high technological literacy tend to favor app-based reminders, which offer more features and a more interactive experience. Healthcare providers need to consider their patients’ demographics and offer diverse reminder options to maximize patient adherence and satisfaction. Combining both modalities (e.g., offering SMS as a basic option and an app as an advanced option) can be an effective strategy.

Digital medication reminders not only help TB patients remember their medication schedules but also play a crucial role in increasing their motivation to remain adherent. This is because TB treatment is very long and often presents psychological challenges [48]. Visualizing the progress and achievements of digital medication reminder apps can increase motivation through tangible and sustainable accomplishments. Many applications use the concept of streaks, which is a series of consecutive days of taking medication. Building and maintaining this streak can be a motivating little game. Some platforms can even award virtual badges or badges for certain achievements, such as completing a month of treatment without missing a dose, which can be a positive psychological boost [52]. Motivation is often strengthened by positive feedback and support. The application can send periodic motivational messages or after the patient successfully takes medication for a certain period. If the PMO, family members, friends, or volunteers are also connected to the app, they can see the patient’s progress and provide direct support, both physically and through text messages. Knowing that someone else cares and is monitoring can be motivating. Digital platforms offer community features or forums where patients can share experiences, ask questions, and receive support from other patients facing similar challenges. Feeling less alone and receiving support from peers can significantly boost motivation [53]. If patients experience severe side effects or other issues that hinder motivation, the app can provide an easy way to communicate with nurses or doctors, ensuring problems are addressed before leading to non-adherence. By combining reminders, positive feedback, community support, and relevant information, digital medication reminders effectively transform the challenging journey of TB treatment into something more manageable and motivating for patients [54].

The findings of this review highlight that technology-based strategies, while effective in supporting TB medication adherence, function primarily as tools that enable and extend nursing practice. Their success is not solely determined by the availability of the technology itself, but by how nurses integrate these tools into holistic, patient-centered care [55]. Nurses play an essential role in conducting initial patient assessments, selecting the most appropriate digital modality based on literacy level and accessibility, providing comprehensive education on treatment and technology use, and offering ongoing therapeutic support [56]. This active involvement ensures that digital reminders and observation systems are not passive interventions, but part of a continuous, adaptive care process tailored to the individual needs of each patient [57].

Furthermore, the effectiveness of these interventions is rooted in the integration of technological solutions within the broader scope of nursing responsibilities, which encompass motivation, psychosocial support, and health education [58]. By leveraging digital platforms, nurses can maintain consistent communication, address treatment-related concerns promptly, and reinforce adherence behaviors through personalized follow-up [59]. This combination of human interaction and technological facilitation reflects a person-centered approach that extends beyond simple reminder functions, ultimately fostering patient empowerment and long-term adherence. Without the skilled involvement of nurses, even the most advanced technological interventions risk becoming impersonal and less effective in addressing the complex barriers to TB treatment adherence [60].

Knowledge is power, and a better understanding of diseases and treatments can be motivating. The application can provide important information about TB, why adherence is important, making it a conscious and motivated decision. With the right information and regular reminders, patients may feel more in control of their condition, reducing anxiety that can erode motivation. When patients feel empowered with information, they are more likely to be active partners in their treatment, rather than just passive recipients. The motivation of TB patients towards digital medication adherence reminders is generally high due to the reminder, monitoring, and social and emotional support features provided. However, privacy and access to technology need to be considered to maintain optimal motivation [54].

## Limitations

Interventions to improve adherence to non-digital medication are excluded. Low-tech strategies are not found in the literature and have been excluded by the search criteria. Studies focused only on digital interventions for TB medication adherence, whereas medication adherence is influenced by a variety of complex socioeconomic, cultural and health system factors, which may not be fully addressed by digital solutions. Article selection bias, where studies tend to select publications that report positive results or successful interventions, ignoring studies that show low effectiveness or failure, thus providing a picture that is not fully representative. The general, non-specific nature of the study area makes it difficult to generalize the findings. Results from one geographical area with specific infrastructure and patient characteristics may not apply in other areas with different access challenges or digital literacy. A limitation of this review is that it only includes English language articles, which may not be representative of nursing interventions in the local context.

## Conclusions

This scoping review demonstrates that nursing interventions integrating digital technology are effective in improving medication adherence among tuberculosis patients. Across the 12 reviewed studies, nurses played key roles in assessing patient needs, selecting appropriate technology (e.g., SMS reminders, mobile applications, video-based directly observed therapy), training patients and their treatment supporters, and conducting ongoing remote monitoring and motivation. By embedding these technologies into nursing practice, nurses can bridge geographical barriers, enhance patient engagement, and support sustained adherence, particularly in remote or resource-limited settings.

Key pillar nursing interventions utilize technology, allowing remote consultation for monitoring, answering questions, and providing support without the need for face-to-face meetings, greatly reducing geographical barriers, and resource efficiency. Innovative methods allow nurses or PMOs to remotely observe patients taking medication asynchronously or synchronously, proving highly effective in ensuring every dose, reducing travel burden, and providing accurate adherence data. Education and motivation through digital media by nurses that is engaging and easy to understand can be implemented to maintain patient morale during long-term treatment. The long-term impact and application of these technology-based nursing interventions have a significant positive impact on long-term care by empowering patients to improve motivation and medication adherence.

The implications of this scoping review suggest that digital-based technologies can be integrated into nursing practice as part of TB treatment management strategies. Nurses can use technology to provide more regular and personalized reminders to patients, and improve remote medication monitoring and support, especially for patients in hard-to-reach areas. Further research on the long-term impact of using this technology on treatment adherence and quality of life for people with TB would be beneficial. To maximize their impact, policymakers and caregivers should pilot scalable, low-cost SMS reminder systems in low-level digital literacy programs for vulnerable populations. Based on the results, it is recommended that nursing protocol interventions include SMS for patients in areas with low digital literacy while VDOT is only provided for urban areas with stable internet.

## Supplementary Information

Below is the link to the electronic supplementary material.

Supplementary Material 1

## Data Availability

All data generated or analyzed during this scoping review are included in this published article.

## Abbreviations

TB: Tuberculosis
MDR-TB: Multidrug-Resistant Tuberculosis
WHO: World Health Organization
DATs: Digital Adherence Technologies
EMMs: Electronic Medication Monitors
VOT: Video-Observed Therapy
VDOT: Video Directly Observed Therapy
SMS: Short Message Service
WOT: Wirelessly Observed Therapy
DOT: Directly Observed Therapy
RCT: Randomized Controlled Trial
JBI: Joanna Briggs Institute
PRISMA: Preferred Reporting Items for Systematic Reviews and Meta-Analyses
HRQoL: Health-Related Quality of Life
EMR: Electronic Medical Record
