# Supplementary material for: Technology-integrated nursing interventions to improve adherence to tuberculosis medication: a scoping review
Source: BMC Nurs. 2025 Sep 2;24:1160. doi: 10.1186/s12912-025-03796-1 (PMC12406418; doi:10.1186/s12912-025-03796-1)
Supplement: Supplementary file 1 — Supplementary Material 1 [file 12912_2025_3796_MOESM1_ESM.docx]

Appendix 1. Search Strategy

| **Database** | **#** | **Search syntax** |
| --- | --- | --- |
| Web of Science | 1 | "Tuberculosis" OR "TB" OR "patients with tuberculosis" |
|  | 2 | "Nursing interventions" OR "nursing care" OR "nursing strategies" OR "nursing practice" |
|  | 3 | "Medical adherence" OR "treatment adherence" OR "compliance" OR "medication adherence" OR "TB treatment compliance" |
|  | 4 | (#1 AND #2 AND #3) |
| Scopus | 1 | ( tuberculosis OR tuberculoses OR mycobacterium AND tuberculosis AND infection ) |
|  | 2 | ( tuberculosis OR tuberculoses OR mycobacterium AND tuberculosis AND infection ) AND TITLE- ABS-KEY ( nursing AND interventions OR evidence AND based ) ) |
|  | 3 | ( tuberculosis OR tuberculoses OR mycobacterium AND tuberculosis AND infection ) AND TITLE- ABS-KEY ( nursing AND interventions OR evidence AND based ) AND TITLE-ABS-  KEY ( medication AND adherence OR patient AND compliance ) ) |
|  | 4 | (#1 AND #2 AND #3) |
| Pubmed | 1 | "tuberculosi"[All Fields] OR "tuberculosis"[MeSH Terms] OR "tuberculosis"[All Fields] OR "tuberculoses"[All Fields] OR "tuberculosis s"[All Fields] OR ("tuberculosi"[All Fields] OR  "tuberculosis"[MeSH Terms] OR "tuberculosis"[All Fields] OR "tuberculoses"[All Fields] OR  "tuberculosis s"[All Fields]) OR ("tuberculosis"[MeSH Terms] OR "tuberculosis"[All Fields] OR ("mycobacterium"[All Fields] AND "tuberculosis"[All Fields] AND "infection"[All Fields]) OR "mycobacterium tuberculosis infection"[All Fields]) |
|  | 2 | "intervention s"[All Fields] OR "interventions"[All Fields] OR "interventive"[All Fields] OR "methods"[MeSH Terms] OR "methods"[All Fields] OR "intervention"[All Fields] OR  "interventional"[All Fields] OR ("evidence based nursing"[MeSH Terms] OR ("evidence  based"[All Fields] AND "nursing"[All Fields]) OR "evidence based nursing"[All Fields] OR  ("evidence"[All Fields] AND "based"[All Fields] AND "nursing"[All Fields]) OR "evidence based nursing"[All Fields]) |
|  | 3 | "medication adherence"[MeSH Terms] OR ("medication"[All Fields] AND "adherence"[All Fields]) OR "medication adherence"[All Fields] OR ("patient compliance"[MeSH Terms] OR ("patient"[All Fields] AND "compliance"[All Fields]) OR "patient compliance"[All Fields]) |
|  | 4 | (#1 AND #2 AND #3) |
